# Tissue Specific DNA Repair Outcomes Shape the Landscape of Genome Editing

**DOI:** 10.3389/fgene.2021.728520

**Published:** 2021-09-03

**Authors:** Mathilde Meyenberg, Joana Ferreira da Silva, Joanna I. Loizou

**Affiliations:** ^1^CeMM Research Center for Molecular Medicine of the Austrian Academy of Sciences, Vienna, Austria; ^2^Institute of Cancer Research, Department of Medicine I, Comprehensive Cancer Center, Medical University of Vienna, Vienna, Austria

**Keywords:** CRISPR-Cas9, genome editing, DNA double-strand break, homology directed repair, non-homologous end-joining, microhomology mediated end-joining, tissue specific DNA repair, tissue stem cells

## Abstract

The use of Clustered Regularly Interspaced Short Palindromic Repeats (CRISPR)-Cas9 has moved from bench to bedside in less than 10years, realising the vision of correcting disease through genome editing. The accuracy and safety of this approach relies on the precise control of DNA damage and repair processes to achieve the desired editing outcomes. Strategies for modulating pathway choice for repairing CRISPR-mediated DNA double-strand breaks (DSBs) have advanced the genome editing field. However, the promise of correcting genetic diseases with CRISPR-Cas9 based therapies is restrained by a lack of insight into controlling desired editing outcomes in cells of different tissue origin. Here, we review recent developments and urge for a greater understanding of tissue specific DNA repair processes of CRISPR-induced DNA breaks. We propose that integrated mapping of tissue specific DNA repair processes will fundamentally empower the implementation of precise and safe genome editing therapies for a larger variety of diseases.

## DNA Double-Strand Break Repair: the Foundation for Genome Editing

Genome stability is constantly challenged by endogenous and exogenous factors that threaten the integrity of DNA. If DNA damage is incorrectly repaired, this leads to mutations or wide-spread genome aberrations that impair cell function and survival. Intracellular reactive oxygen species (ROS) and reactive nitrogen species (RNS), reactive metabolites, and replication stress synergise with exogenous genotoxic sources of damage, such as radiation, chemical exposure, viral, or bacterial infections to challenge genomic stability. In order to protect genome integrity, cells have evolved sophisticated mechanisms to detect, signal, and repair diverse DNA lesions, known as the DNA damage response.

### Biological Significance of DNA Double-Strand Breaks

DNA double-strand breaks (DSBs) are amongst the most toxic lesions cells can encounter, as both DNA ends become topologically separated. For this reason, DSBs are induced in cancer therapy, either through ionising radiation or by preventing their repair *via* topoisomerase inhibition. In contrast, formation of endogenous DSBs is an integral part of fundamental cellular processes, such as the generation of immune receptor diversity, meiosis, and ageing (Jackson and Bartek, 2009). Therefore, DSB repair is an essential and vital cellular process. Overall, DSBs are repaired in two ways: re-ligation of the DNA ends through pathways such as non-homologous end-joining (NHEJ) and microhomology-mediated end-joining (MMEJ), or templated repair from a separate donor DNA molecule, through a process called homology directed repair (HDR; Yeh et al., 2019). A key aspect in the repair of DSBs in human cells is the competition between these two types of repair, with end-joining pathways being favoured over templated repair, in a cell-cycle dependent manner.

### Cas9-Induced DNA Double-Strand Breaks: The Genome Editing Revolution

During the early 2000s, site-specific DSB generation, induced by engineered endonucleases, became an increasingly useful approach to edit the genome. Zinc finger nucleases (ZFNs) and transcription activator-like effector nucleases (TALENs) have been successfully used as genome editing tools in mammalian cells (Miller et al., 2011; Hossain et al., 2015). However, inherent difficulties with protein design, synthesis, and validation remained a challenge to the widespread implementation of these nuclease-based editing technologies. This limitation was solved upon the discovery of Clustered Regularly Interspaced Short Palindromic Repeats (CRISPR), a breakthrough that revolutionised the field of genome editing (Jinek et al., 2012). CRISPR and the associated Cas9 endonuclease (CRISPR-Cas9) were initially identified as an antiviral defence mechanism in prokaryotes, but rapidly became a powerful genome editing tool in eukaryotic cells (Cong et al., 2013; Jinek et al., 2013; Mali et al., 2013). The CRISPR-Cas9 system, guided by a single-guide RNA (sgRNA), targets a particular region of the genome, generating a DNA DSB that subsequently activates the cellular DNA repair machinery. The considerable ease of manipulating the sgRNA, compared to ZFNs and TALENs, has served an important role in the CRISPR revolution, creating the possibility to edit a wide variety of cell types and organisms, with unprecedent precision and efficiency. Importantly, besides being a powerful approach for functional genetic studies, CRISPR-Cas9 approaches hold great promise for the correction of genetic disorders caused by specific alterations in the genome, with recent clinical trials reporting promising results (Wang et al., 2020; Frangoul et al., 2021). However, most current clinical applications are still based on the disruption of a genetic sequence, rather than a precise edit. Moreover, the safety and efficiency of CRISPR-based therapies still need to be closely addressed and an important step is the fundamental understanding of the tissue specific DNA repair pathway choice, following a Cas9-induced DSB. The focus of this review will be on the DSB-dependent genome editing technologies which make use of *Streptococcus pyogenes* Cas9 (SpCas9), generating a blunt end at a targeted genomic site. We direct readers to the following additional technical advances that have expanded the CRISPR-toolbox and fall outside the focus of this review: engineered Cas9 nucleases with higher fidelity (Kleinstiver et al., 2016) and broader specificity (Kleinstiver et al., 2015; Walton et al., 2020), DSB-independent applications that increase the range of possible editing outcomes, such as DNA base editors (Komor et al., 2016; Gaudelli et al., 2017) and prime editing (Anzalone et al., 2019), CRISPR-mediated regulation of gene expression (Gilbert et al., 2013; Qi et al., 2013; Nuñez et al., 2021), and new CRISPR nucleases repurposed for genome editing (Zetsche et al., 2015).

## Repair of Cas9-Induced DNA Double-Strand Breaks

### Cell Cycle Regulates DNA Double-Strand Break Repair Pathway Choice

After a Cas9-induced DSB, repair pathway choice is a crucial factor in determining the editing outcome. The blunt ends of the DNA break can be protected by the Ku70/80 heterodimer, fating the lesion for repair by NHEJ. Conversely, 5'–3' resection of DNA ends reveals sequence homologies that direct repair toward HDR or MMEJ (Yeh et al., 2019). Therefore, the processing of DSB ends from blunt ends to overhangs, *via* end-resection, is the major factor dictating repair pathway choice. Although HDR faithfully repairs lesions, the end-joining pathways are preferentially upregulated through several mechanisms following DSB formation. This is because NHEJ is active throughout all phases of the cell cycle, predominating in G0 and G1 (Shrivastav et al., 2008), whereas factors that promote extensive end-resection are more active during S and G2 phases, favouring HDR when a sister chromatid is present (Chang et al., 2017). The balance between HDR and NHEJ is further regulated by reciprocal inhibition between these two pathways. While 53BP1 and RIF1 mostly promote NHEJ by blocking end-resection, BRCA1 and CtIP direct break processing toward HDR or MMEJ (Escribano-Díaz et al., 2013).

### End-Joining Repair

**Figure 1:**
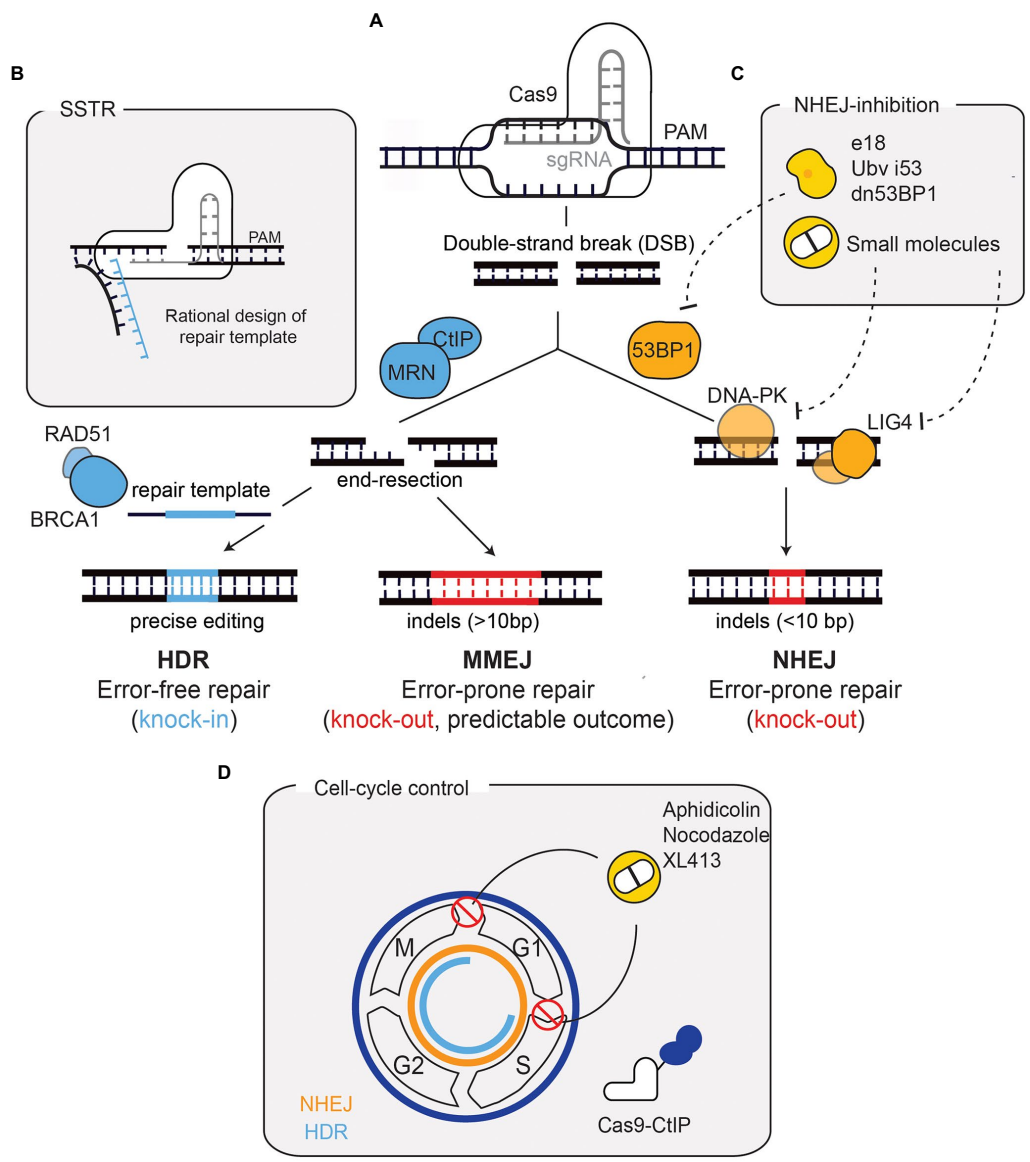
Repair outcomes after a Cas9-induced DNA double-strand break (DSB) and strategies for enhancing precise repair. **(A)** Cas9, targeted by a sgRNA, induces a DSB in a precise region of the genome. Non-homologous end-joining (NHEJ), promoted by 53BP1, is the default repair pathway. Through the coordinated action of factors such as DNA-PK and LIG4, NHEJ repairs the DSB by re-joining the DNA-ends in an error-prone manner. This results in small insertions and deletions (indels) that can generate a loss-of-function allele if a frameshift is generated. If end-resection occurs [mediated by CtIP and MRE11-Rad50-NBS1 (MRN)], microhomology-mediated end-joining (MMEJ), or homology-directed repair (HDR) function. The repair outcome following MMEJ-mediated repair can vary, although this can be predicted since it depends on regions of microhomology and leads to larger indels. HDR, mediated by factors such as BRCA1 and RAD51, relies on a repair template and hence is error-free, leading to precise genomic alterations. **(B)** The use of ssDNA oligonucleotides (ssODN) as donor templates has also been developed to harness HDR. This process is called single-stranded templated repair (SSTR). SSTR is generally more efficient due to the asymmetry of the Cas9-DNA complex, which leads to the release of the PAM-distal non-target strand. Therefore, a rational design of the ssODN donor template complementary to the strand that is first released improves precise editing. **(C)** The inhibition of NHEJ has been used to improve precise repair following Cas9-breaks. 53BP1 inhibition through ubiquitin variants, dominant negative forms, or expression of factors that displace 53BP1, has proven useful. Small molecule inhibitors against DNA-PKcs and LIG4 have also been used. **(D)** Cell cycle manipulation has also proved useful for enhancing HDR. HDR (depicted in blue) is only active in S/G2/M phases, contrary to NHEJ (depicted in orange), which is active throughout the cell cycle. Strategies to improve HDR have included the use of compounds (such as XL413, aphidicolin, and nocodazole) to block cells in HDR-permissive phases. A Cas9-CtIP fusion allows end-resection (and subsequently HDR) to occur throughout the entire cell-cycle. PAM, protospacer adjacent-motif.

In the absence of a repair template, a Cas9-induced DSB is predominantly repaired in an error-prone manner, resulting in insertions and deletions (indels) within the targeted genomic sequence. If these indels give rise to frameshift mutations, they result in loss-of-function alleles. This type of repair outcome has been largely attributed to the use of NHEJ, which directly ligates the two DNA ends following cleavage, leading to the generation of small indels (<10bp; Bothmer et al., 2017). More recently, MMEJ has been shown to contribute to a large fraction of the edited alleles observed after genome editing (Shen et al., 2018). The MMEJ-mediated repair of Cas9-induced DSBs is characterised by a distinct indel profile where larger deletions are the predominant outcome (>10bp; Ferreira da Silva et al., 2019; Figure 1A). Similar to NHEJ, MMEJ ligates the DNA ends in the absence of an exogenous repair template but, unlike NHEJ, MMEJ requires initial and short-distance DSB end-resection to reveal regions of microhomology (Seol et al., 2018). The initial resection (5–25 base pairs) is performed by the MRE11-Rad50-NBS1 (MRN) complex, which is activated in a cell-cycle dependent manner by CtIP (Truong et al., 2013). This exposes microhomologies on opposite strands that anneal to one another. DNA polymerase θ (POLQ) stabilises the annealed single-stranded DNA and fills the gaps, *via* templated synthesis. The early resection steps that occur in MMEJ are shared with HDR. However, annealing and extension of overhanging ends during MMEJ function to prevent HDR. Moreover, HDR requires extended end-resection, which depends on additional factors, such as the helicase Bloom syndrome protein (BLM) and Exonuclease 1 (EXO1; Truong et al., 2013).

Albeit being generally considered as an alternative pathway, studies based on the pharmacological and genetic ablation of NHEJ have shown that MMEJ can fully compensate for the absence of NHEJ in the repair of Cas9-induced DSBs (Brinkman et al., 2018; Ferreira da Silva et al., 2019). Despite the error-prone nature of end-joining pathways, there is mounting evidence indicating that the pattern of DNA repair following a Cas9-induced DSB is not stochastic (van Overbeek et al., 2016; Shou et al., 2018). Based on this observation, several studies have systematically analysed how sequences flanking the DSB impact repair outcome, leading to the important conclusion that template-free Cas9 editing can be predicted and applied to achieve a specific outcome (Allen et al., 2018; Shen et al., 2018).

### Homology-Directed Repair

In contrast to the end-joining pathways, and within the context of genome editing, HDR depends on an exogenous repair template, allowing cells to integrate specific and precise alterations in their genome (Figure 1A), thus making it more relevant for therapeutic applications. HDR efficiency, however, remains a challenge and several approaches have been developed to overcome this limitation. Biochemical modelling of the Cas9-DNA interaction has been fundamental to prove that the efficiency of HDR can be improved through rational design of the repair template, concluding that the use of single-stranded DNA (i.e., synthetic oligonucleotides) as a repair template improves HDR (Richardson et al., 2016; Aird et al., 2018). This sub-type of HDR is commonly called single-stranded templated repair (SSTR; Figure 1B).

Importantly, transcriptional and genetic differences impact the efficiency of CRISPR-Cas9 editing and therefore the effectiveness of genome editing approaches. Screens performed in human cancer cell lines have shown that the Fanconi anaemia (FA) pathway diverts repair toward SSTR, playing an important role in HDR efficiency (Richardson et al., 2018). The Fanconi anaemia group D2 protein (FANCD2) has been shown to have a direct role on genome editing, by physically localising to Cas9-induced DSBs. This finding has important therapeutic implications for future genome editing applications in FA patients. Moreover, the involvement of FA, a pathway that repairs interstrand cross-links, on the repair of Cas9-mediated DSBs highlights how little is known about the interplay between DNA repair pathways in the context of different CRISPR-mediated technologies.

### Rewiring DNA Double-Strand Break Repair Towards Homology-Directed Recombination

The importance of DNA repair for genome editing applications is further illustrated by the different approaches that modulate DNA repair pathways to improve HDR efficiency. For example, since NHEJ is the default pathway in human cells, its inhibition has been exploited to favour HDR. This has been achieved through the use of small-molecules targeting LIG4 or DNA-PKcs (Robert et al., 2015; Riesenberg and Maricic, 2018), ubiquitin-variants targeting 53BP1 (Canny et al., 2017), expression of factors that displace 53BP1 from DSBs (Nambiar et al., 2019), or 53BP1 dominant negative forms (Paulsen et al., 2017; Figure 1C). Another strategy to promote HDR is through cell cycle modulation, thereby increasing precise editing and minimising undesirable indels (Figure 1D). One of such strategies makes use of a Cas9 fused with the protein CtIP (Charpentier et al., 2018). This construct bypasses the requirement for cell cycle dependent activation of CtIP (by CDK1/2), necessary for end-resection and subsequent HDR. Pharmacological cell cycle arrests in HDR-permissive phases (S/G2) with aphidicolin, nocodazole, or the small molecule XL413, can also improve the efficiency of precise editing (Lin et al., 2014; Wienert et al., 2020). Overall, the modulation of DNA repair pathway choice, either through direct inhibition of NHEJ or cell-cycle regulation, comprises a potent strategy to boost precise editing.

### CRISPR-Cas9 Editing Outcomes Are Shaped by DNA Repair Processes

The DNA damage response is a highly interconnected signalling network, which is modulated by cell cycle stage, gene expression changes, chromatin states, differentiation status, and cell type (Blanpain et al., 2011; Fortini et al., 2013; Klement and Goodarzi, 2014; Polak et al., 2015; Hustedt and Durocher, 2017; Weeden and Asselin-Labat, 2018; Yimit et al., 2019).

In the pursuit of safe and precise genome editing, next generation sequencing (NGS) technologies have empowered researchers to look for off-target effects beyond commonly predicted sites, enabling high standards for quality control of *ex vivo* edited cell populations (Li et al., 2019). Even in the near absence of off-target editing, the challenge of achieving precise editing outcomes at the desired target site remains. Investigating CRISPR-Cas9 outcomes in mouse embryonic stem cells, mouse hematopoietic progenitors, and differentiated human cells lines with intact DNA repair, Kosicki et al. (2018) found frequent large-scale deletions around the cut site, as well as crossover events with distant sites. Notwithstanding the advanced technologies to limit off-target effects, these surprising results revealed that more research is required to understand possible editing outcomes and how to avoid unwanted on-target effects.

A recently developed approach termed Repair-Seq was used to systematically map DNA repair outcomes, and hence editing outcomes, after Cas9 and Cas12a mediated genomic editing across several loci (Hussmann et al., 2021). This revealed that genetic dependencies driving repair outcomes are determined by the exact type of DNA lesion present. Predicting editing outcome is thus dependent on the understanding of lesion conformation and its interplay with DNA repair factors.

In summary, recent insights into the complex interplay between DNA break configuration and DNA repair factors, highlighted how the landscape of genome editing outcomes remains underexplored. The studies discussed above made their observations in a few cellular models but found a surprising variety of lesions and repair outcomes generated. The level of complexity further increases when one takes cell type and tissue specific effects of DNA repair into consideration. It becomes apparent that the full control of CRISPR-mediated genome editing is only possible with full understanding of the intricacy of endonuclease generated lesion conformation in combination with DNA repair regulation in a tissue dependent context.

## Success of Crispr-Based Therapies Depends on Understanding Tissue Specific DNA Repair

### DNA Repair Outcomes Are Tissue Specific

Outside the CRISPR field, it has long been noted that the balance between the type of DNA lesion and DNA repair activity determines tissue specific repair outcome. Germline mutations in DNA repair genes cause disease phenotypes, which often manifest in a tissue specific manner. A classic example are *BRCA1/2* mutations, which cause a defect in HDR, yet predispose primarily to breast and endometrial cancers. Similarly, defects in DNA single strand break repair (SSBR), predominantly affect neuronal cell types, while, for instance defects in crosslink repair (Fanconi anaemia pathway) precipitate bone marrow failure and neurological degeneration (Tiwari and Wilson, 2019). The differential effect certain DNA repair defects have on specific cell types cannot be fully explained. Part of the explanation may be tissue specific differences in terms of which type of DNA damage is encountered, for instance, due to differential cellular metabolism or hormone levels (Langevin et al., 2011; Garaycoechea et al., 2012; Singh and Yu, 2020). However, DNA damage is only one side of the coin, while DNA repair is the other. Indeed, different cell types, even within tissues, have been found to show divergent propensity for DNA repair. Differential sensitivity to DSBs, for instance, has been observed among human hematopoietic stem cells (HSCs) and progenitor cell populations (Milyavsky et al., 2010). Compared to progenitor populations, HSCs showed delayed repair kinetics and higher levels of p53 activation, leading to increased apoptosis after DSB induction.

How the cell type affects the specificity of DNA repair outcomes across tissues is thus another level of consideration for designing CIRSPR applications. Although the intricate tissue specific response to DNA DSBs complicates design of gene editing therapies, in-depth characterization of tissue specific DNA repair mechanisms is key for developing safe and efficient therapies. We discuss recent insights which advanced the understanding of underlying mechanisms effectuating tissue specificity of DNA repair, and how this might influence CRISPR applicability.

### Tissue Specific Cell Cycle Effects

Since cell cycle stage impacts repair pathway choice, only actively cycling cells have full accessibility to NHEJ, MMEJ, and HDR. Other cells, quiescent or post-mitotic, must re-enter the cell cycle to access DSB repair and other repair pathways (Nouspikel and Hanawalt, 2000; Shin et al., 2020). Upon exit of G0, NHEJ is the predominant repair pathway for DSBs, increasing the possibility of mutagenic repair (Mohrin et al., 2010; Shin et al., 2020). The inaccessibility of HDR coupled with the preference for NHEJ in some cell types poses a problem for the utility of CRISPR therapeutics. To achieve a long-lasting therapeutic effect, targeting long-lived stem cell populations offers the best strategy. However, many somatic stem cells across tissues are quiescent and therefore HDR-based therapies aimed at introducing specific edits are challenging and might limit the applicability of CRISPR technology in the clinics. A recent study, however, has demonstrated that detailed knowledge of DNA repair and cell cycle regulation can significantly increase the HDR-editability of the target cell population. Shin et al. demonstrated that quiescent HSCs can be edited with HDR up to an overall efficiency of 30% if they are stimulated to enter the cell cycle before commencing editing.

### Tissue Specific Effects of Differentiation and Chromatin Status

It has been established that many different cell lineages across tissues exhibit slower rates of DNA repair and generally have reduced capacity to maintain their genome. This can be seen as an adaptive advantage, as highly differentiated cells do not spend energy on whole genome maintenance and instead focus on the conservation of actively transcribed genes (Nouspikel and Hanawalt, 2002). Most terminally differentiated cells are not of interest for CRISPR therapeutics, apart from long-lived differentiated cells such as neurons and intermittently mitotic hepatocytes. For the most part, tissue specific stem cells will be the target for clinical CRISPR applications by virtue of their ability to populate the tissue with gene-edited cells. Because DNA repair, from signalling to pathway choice, is tightly interconnected with epigenetic regulation, it must be appreciated that the distinct chromatin profiles of differentiated and non-differentiated cells might influence how a DNA lesion is repaired. HDR, in contrast to NHEJ, requires end-resection, which happens more effectively in open chromatin regions. Consequently, HDR is favoured in genomic regions with open chromatin conformation, marked by H4 acetylation and HeK36me3. NHEJ, on the other hand, is preferred in heterochromatic regions and at sites where H4 is demethylated at lysine 20 (H4K20me2; Karakaidos et al., 2020). Recently, the pathway balance between NHEJ and MMEJ as influenced by chromatin configuration has also been mapped (Schep et al., 2021). This study showed that MMEJ is more active than NHEJ in specific heterochromatin contexts, namely late replicating regions, lamina associated regions, and at H3K9me2 sites. Moreover, MMEJ was shown to compete with SSTR (Schep et al., 2021). Therefore, systematically mapping chromatin environments across cell types can inform avenues for regulation to successfully install CRISPR edits which rely on the incorporation of repair templates.

The advances in mapping and understanding intrinsic differences in DNA repair regulation across cell types will undoubtedly promote design of more efficient CRISPR therapies, which can be applied *ex vivo* using induced pluripotent stem cells (iPSCs) and organoid-based approaches (Schwank et al., 2013; Xie et al., 2014; Li et al., 2015), while keeping unwanted on-target effects to a minimum. Especially when targeting long lived and actively dividing stem cells, *ex vivo* editing offers a safer route over *in vivo* editing, because edited cells can be thoroughly investigated and selected for the desired editing outcome, prior to transplantation into the patient. However, some diseases may require *in vivo* editing due to the plurality of tissues and cell types affected, adding another layer of complexity, since tissue context must be considered as well.

### Editing Outcomes Are Influenced by Tissue Architecture

One disease in which *in vivo* editing would likely be necessary is cystic fibrosis, which is caused by mutations in the cystic fibrosis transmembrane conductance regulator (*CFTR*) gene. The function of this chloride/bicarbonate channel is to regulate the exchange of electrolytes and thus the hydration levels of secretory epithelia. Loss or reduction of function in this protein leads to cycles of mucus accumulation, inflammation, and infection in the lung, progressively destroying the airway epithelium (Ensinck et al., 2021).

With 360 reported pathogenic mutations, editing strategies for cystic fibrosis need to be tailored to each patient and draw on an integrated understanding of DNA repair. In order to achieve a long-term cure, the resident tissue stem cells, i.e., basal cells, must not only be studied in terms of their response to CRISPR-induced DNA breaks and subsequent repair, but also where they are situated within their host tissue. This is especially relevant because, within the lung, an intra-tissue variance in response to DNA damage exists. Along the airway epithelium of the trachea and larger bronchi, basal stem cells are responsible for renewing the epithelium, giving rise to ciliated and club cells (Rock et al., 2009; Asselin-Labat and Filby, 2012; Hogan et al., 2014). It should be noted that basal cells are the most active stem cell pool along the trachea, whereas in the bronchi, club cells have also been shown to self-renew and give rise to ciliated cells (Rawlins et al., 2009). Within the lung tissue, there is also the highly specialised alveolar epithelium, which consists of elongated type 1 cells and secretory type 2 cells (alveolar type 2=AT2), the latter being the resident stem cell (Barkauskas et al., 2013; Yamamoto et al., 2020). Surprisingly, it has been observed that basal stem cells exhibit a greater capacity for repair of DSBs compared to AT2 cells. Basal cells utilise NHEJ more efficiently than AT2 cells, allowing them to resist apoptosis and to begin proliferation. In the disease context, the pathologic changes and inflammatory environment of the tissue also play a role in how efficient CRISPR editing might function. Hence, to avoid a mixture of editing outcomes across different cell types within one tissue, the utilisation of DNA repair pathways and their relative efficiency in the target cells must be taken into consideration for CRISPR-Cas9 editing.

As the CRISPR field advances, it has become ever increasingly interwoven with the DNA repair field, because it is recognised that genome editing is dependent on the activity of the cellular DNA repair machinery. We focused on CRISPR-Cas9 technologies, which depend on DSB repair pathways and reviewed the emerging research on the complexity of tissue specificity of DNA repair. The outcome of a genomic edit builds upon the complex interplay of the DNA repair machinery, which is specific to the type of lesion generated, and differs across cell types and within tissue environments, owing to cell cycle effects, differentiation status, and chromatin configurations. The power to translate genome editing to the clinic increases with a progressive understanding of all aspects of DNA repair.

## CRISPR in the Clinics: Challenges and Limitations Due to DNA Repair Tissue Specificity

With ever improving CRISPR-based technologies, gene-editing treatment has become a reality in the clinics. The dream to cure diseases by correcting the causative mutations is far simpler than its implementation. For a few applications, including engineering T-cells for cancer therapy, inborn blood disorders, transthyretin (TTR) amyloidosis, and heritable blindness, CRISPR-therapies have become available to patients. We review recent achievements in clinical trials and consider the applicability of tissue specific DNA repair.

### CRISPR in Cancer Therapy

Recently concluded clinical trials have successfully shown delivery of CRISPR-Cas9-based *ex vivo* therapies to patients and demonstrated safety and feasibility of these treatments. Yet, these trials have also demonstrated that the mere reduction of off-target editing is not sufficient to achieve the desired outcome. One trial (NCT02793856) studying the therapeutic effect of knocking out the programmed cell death protein 1 (PD-1) in patient derived T-cells *via* NHEJ in refractory non-small-cell lung cancer, found a good ratio of 48.7 of on-target over off-target editing. Even so, 28.8% of all on-target edits did not match the predicted outcome (Lu et al., 2020). Another trial (NCT03399448), also focused on enhancing anti-tumor immunity of T-cells, set out to simultaneously edit four loci encoding for the endogenous T-cell receptor (TCR), and PD-1, while introducing a transgene (NY-ESO-1), which is more efficient at recognising tumor cells than the TCR. While off-target editing events were rare, simultaneous editing of multiple loci led to translocations and large deletions. Of 12 possible translocation events, the most abundant rearrangement caused a 9.3kb deletion, which was evident in all edited samples and remained detectable in patients up to 170days post-transfusion (Stadtmauer et al., 2020). While all observed translocations persisted in peripheral blood, the frequency of detected rearrangements declined with time, indicating no specific growth advantage introduced by the unintended edits.

In summary, both trials demonstrated the utility of CRISPR-Cas9 based treatment approaches in patients, in addition to moderate clinical benefit. The editing strategy in both trials minimised off-target effects, while still introducing unwanted on-target effects. For transient cell populations such as engineered T-cells, this might be acceptable. However, for clinical applications which require precise editing of resident stem cell populations, better control over editing outcome is needed.

### CRISPR for Hereditary Disease Therapy

#### Targeting Tissue Stem Cells

**Figure 2:**
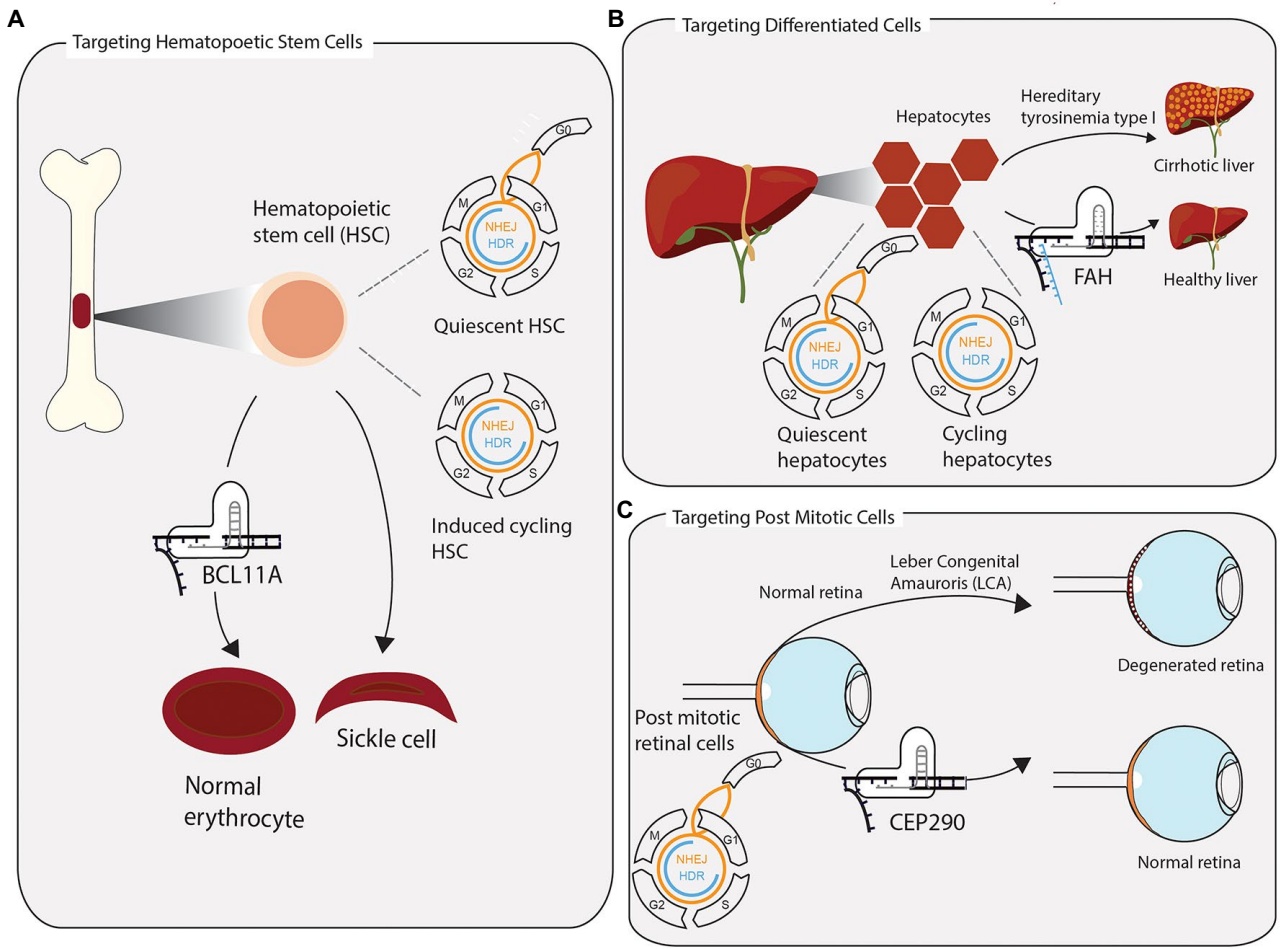
Successful clustered regularly interspaced short palindromic repeats (CRISPR) applications require consideration of tissue-specific DNA repair and repair pathway accessibility. **(A)** Hematopoietic stem cells (HSCs) are extracted from the bone marrow and edited *ex vivo* for the treatment of sickle cell anaemia and β-thalassemia. Without stimulating cells to enter the cell cycle once before CRISPR-Cas9 editing, quiescent HSCs rely on error prone NHEJ to repair induced DSB. In a clinical application, the preference for NHEJ is leveraged to disrupt the transcription factor BCL11A, which represses the expression of foetal hemoglobin. The re-expression of foetal hemoglobin allows for the formation of normally shaped erythrocytes. **(B)** The toxic accumulation of fumarylacetoacetate in fatal hereditary tyrosinemia type I (HTI) leads to liver cirrhosis and liver failure due to a mutation in the fumarylacetoacetate hydrolase gene (FAH). Highly differentiated quiescent cells can be stimulated to re-enter the cell cycle upon DNA, or tissue, damage. Provided with a single-stranded repair template, few cycling hepatocytes have access to repair DSB *via* homologous directed repair. Precisely edited hepatocytes have a growth advantage over non-edited cells and reconstitute tissue homeostasis. **(C)** Leber congenital amauroris (LCA) is the first disease treated with an *in vivo* CRISPR approach. The post mitotic light sensitive cells in the retina degenerate with age, leading to impaired vision early on in life. Appropriating the propensity of post mitotic cells to repair DSBs *via* NHEJ, the therapy aims to disrupt an aberrant splicing site in exon 26 of CEP290, maintaining a functional retina.

An important milestone in the development of therapeutic genome editing was reached in two CRISPR-based trials for β-thalassemia and sickle cell anemia (NCT03655678 and NCT03745287, respectively). Targeting CD45-positive hematopoietic stem and progenitor cells, the *ex vivo* editing strategy relied on error prone NHEJ to achieve gene knockout of *BCL11A*, a transcriptional repressor of foetal hemoglobin (Frangoul et al., 2021). Precise correction of the causative point mutations for these diseases seems like a more obvious choice compared to disrupting a transcription factor (Figure 2A). However, considering the relative ineffectiveness of HDR in the target cells and their propensity to utilize NHEJ, deliberate indel generation offers a more effective editing strategy. Both trials proved that minimising off-target effects, while carefully predicting and evaluating indels generated at the on-target site, are valid strategies to utilise NHEJ for safe editing of stem cells. Edited cells engrafted in patients’ bone marrow, demonstrating the feasibility of editing long lived stem cells and replenishing stem cell compartments of interest with corrected cells (Frangoul et al., 2021). In future applications, which require precise editing, controlling quiescent and cycling states of HSCs might prove useful to increase HDR (Shin et al., 2020).

#### Targeting Differentiated Cells

Integrated knowledge of tissue architecture and DNA repair outcomes can help designing better CRISPR therapies. A prime example of this is the fatal genetic disease hereditary tyrosinemia type I (HTI). HTI is caused by a G>A point mutation in the fumarylacetoacetate hydrolase (FAH) gene, which causes skipping of exon 8, leading to a dysfunctional protein and accumulation of the toxic metabolite fumarylacetoacetate in hepatocytes, ultimately leading to cirrhosis, acute liver failure, and increased risk of hepatocellular carcinoma (Yin et al., 2014; King et al., 2017). The liver consists largely of highly differentiated hepatocytes, while the population of hepatic progenitor cells (HPCs) is considerably smaller. Although fully differentiated, in response to disturbances to homeostasis, quiescent hepatocytes can enter the cell cycle and begin proliferating to repair tissue injury (Figure 2B; Kiseleva et al., 2021). In their study on HTI, Yin and colleagues demonstrated that precise correction of the mutation can be achieved in mice *via* delivering CRISPR-Cas9 along with a single-stranded DNA repair template into hepatocytes, using hydrodynamic tail vein injection. Once stimulated to proliferate, actively cycling hepatocytes can utilize HDR to make the edit of interest. Although only one in 250 liver cells were successfully edited, corrected cells have a selective advantage and begin to outgrow unedited cells and repopulate the liver, effectively ameliorating the disease. Therefore, considering tissue architecture along with DNA repair pathway choice, results in a therapy which is more effective than the initial editing efficiency.

Gene editing of hepatocytes has recently found application in a clinical trial using *in vivo* editing (Gillmore et al., 2021). TTR amyloidosis (ATTR) is a progressive fatal disease, which may be inherited in an autosomal dominant manner through inheritance of one of more than 100 recognised pathogenic mutations in the TTR protein. Misfolding of mutant TTR promotes the accumulation of insoluble protein fibers, which are deposited predominantly in heart and nervous tissue, leading to cardiomyopathies and polyneuropathies. TTR has normal, but dispensable, functions in vitamin A transport and is almost exclusively produced in the liver. Thus, targeted knockout of the TTR gene in hepatocytes, coupled with vitamin A supplementation, is a viable treatment strategy to reduce systemic levels of TTR and curb the deposition of pathogenic TTR fibers (Gertz et al., 2015).

Gillmore et al. (2021) describe the intermediate results of an ongoing clinical study seeking to reduce TTR protein level in patients with hereditary ATTR (Gillmore et al., 2021). Extensive pre-clinical screening for off-target effects was conducted to allow for the optimal selection of an efficient sgRNA and the formulation of the editing drug “NTLA-2001.” The CRISPR editing machinery, encoded in mRNA, and the TTR sgRNA was delivered encapsulated in lipid nanoparticles with liver tropism. Patients showed a dose dependent effect of TTR serum level reduction after 28days, between 47–56 and 80–96% for the lower and higher dose of NTLA-2001, respectively. Thus far, patients have not exhibited serious adverse effects. Long-term monitoring of protein level reduction, side effects, and outcomes on disease progression and mortality will show the safety and applicability of this therapy. The liver is an optimal target organ for the first *in vivo* therapy targeting differentiated cells. It consists mostly of intermittently mitotic hepatocytes, which at once reduces the risk of pathogenic outgrowth, compared to consistently cycling cells, and simplifies the complexity of having to consider many cell types in the design of the editing strategy. Aside from the rarity of hereditary ATTR, pathogenic accumulation of wild type TTR fibers in the heart is also observed in patients and has been recognised as a cause for cardiomyopathy and eventual heart failure (Gertz et al., 2015). Hence, a successful CRISPR therapy for transthyretin amyloidosis may be the first to find broad application beyond rare diseases.

#### Targeting Post Mitotic Cells

Since specificity of editing outcomes and safety are still major technological hurdles, there are currently few ongoing clinical trials utilising *in vivo* CRISPR Cas9 editing. One trial is seeking to treat Leber congenital amaurosis (LCA; ClinicalTrials.gov, 2019). LCA manifests in degeneration of the retina and is caused by mutations in more than 25 genes (Daich Varela et al., 2021). The CRISPR-based drug, EDIT-101, targets a heterozygous mutation in intron 26 of the LCA gene CEP290 to remove an aberrant splicing site *via* generating an indel through NHEJ (Figure 2C; Maeder et al., 2019). While it is exciting that *in vivo* CRISPR editing begins to move into the clinic, it is pertinent to keep in mind that LCA constitutes an ideal model disease for this approach. The post-mitotic nature of the targeted cells ensures a greater propensity for utilising NHEJ to repair the induced break and reduces the risk of selective pathogenic outgrowth of edited cells, when compared to actively cycling somatic stem cells. Furthermore, there is reduced risk of inflammation or adverse reactions to introduction of Cas9, due to the immunoprivileged status of the eye.

The examples above illustrate the potential and versatility of CRISPR-based therapies. The success of such approaches, however, relies on careful consideration about the biology of targeted cells and a deep understanding about the tissue specific mechanisms of DNA damage signalling and repair.

## Concluding Remarks and Future Perspectives

The successful implementation of CRISPR-Cas9 technologies in a clinical setting relies on a deeper understanding of the DNA repair mechanisms and pathways responsible for genetic replacement outcomes, as well as the activity and accessibility of these pathways in specific cell types and tissues. Following the generation of a DSB, cell cycle regulation, and DNA repair pathway choice play major roles in determining the editing outcome. Therefore, genome editing approaches have begun to harness DNA repair control and modulation for more efficient and predictable outcomes.

Overall, the genome and transcriptome of target cells impact the effectiveness of genome editing approaches. Moreover, cell identity and tissue context are important considerations in designing effective editing strategies. While *ex vivo* editing strategies allow for extensive quality control, *in vivo* editing strategies could target multiple cell types at once, but must be safe and accurate, especially when targeting long-lived somatic stem cells. Recent successes in therapeutic editing achieved in β-thalassemia and sickle cell anemia demonstrated the feasibility of utilising CRISPR-Cas9 editing in stem cells to alleviate disease. While these reports are encouraging, there is a large margin for improving treatment strategies for diseases which require editing of multiple loci or precise editing of one locus across multiple tissues. CRISPR technologies that do not rely on the generation of DSBs, such as DNA base editors and prime editing, are promising avenues for future precision medicine. These technologies are independent of cell cycle stage and hence have the potential to correct multiple cell types. However, both base editors and prime editing introduce unique types of DNA damage products, such as DNA single-strand breaks and base mismatches, to facilitate genome editing. Hence these approaches rely on other DNA repair pathways that must be understood, in tissue-specific contexts, for further expansion and improvement of these technologies (Gu et al., 2021).

The expansion of the tools available to understand and control the CRISPR-Cas9 system has continuously fuelled the development of new therapeutic strategies and has brought a fundamental discovery into the clinics in less than a decade. The implications for personalised medicine are immense. However, for this steep trajectory to continue and to broaden the applicability and impact of these technologies, the focus of future developments must shift to include the investigation of tissue specific DNA repair. Knowledge of the underlying mechanisms of how the DNA repair machinery reacts to a CRISPR break within a distinct cellular context is a key to mapping the landscape of genome editing.

## Author Contributions

MM and JF wrote the first draft of the manuscript. All authors contributed to the article and approved the submitted version.

## Conflict of Interest

The authors declare that the research was conducted in the absence of any commercial or financial relationships that could be construed as a potential conflict of interest.

## Publisher’s Note

All claims expressed in this article are solely those of the authors and do not necessarily represent those of their affiliated organizations, or those of the publisher, the editors and the reviewers. Any product that may be evaluated in this article, or claim that may be made by its manufacturer, is not guaranteed or endorsed by the publisher.
